# Laser-induced fluorescence in malignant and normal tissue in mice injected with two different carotenoporphyrins.

**DOI:** 10.1038/bjc.1994.413

**Published:** 1994-11

**Authors:** H. Nilsson, J. Johansson, K. Svanberg, S. Svanberg, G. Jori, E. Reddi, A. Segalla, D. Gust, A. L. Moore, T. A. Moore

**Affiliations:** Lund University Medical Laser Center, Sweden.

## Abstract

Laser-induced fluorescence (LIF) was used to characterise the localisation of an intravenously administered trimethylated carotenoporphyrin [CP(Me)3] and a trimethoxylated carotenoporphyrin [CP(OMe)3] in an intramuscularly transplanted malignant tumour (MS-2 fibrosarcoma) and healthy muscle in female Balb/c mice, 3, 24, 48 and 96 h post injection. The fluorescence was induced with a dye laser pumped by a nitrogen laser, emitting light at 425 nm. The fluorescence spectra were recorded in the region 455-760 nm using a polychromator equipped with an image-intensified CCD camera. The tumour/peritumoral muscle ratio was about 5:1 for CP(Me)3 and about 6:1 for CP(OMe)3 in terms of the background-free fluorescence intensity, which peaked at about 655 nm. By including the endogenous tissue fluorescence, the contrast was further enhanced by a factor of approximately 2.


Br. J. Cancer (1994), 70, 873-879 © Macmillan Press Ltd., 1994

### Laser-induced fluorescence in malignant and normal tissue in mice injected with two different carotenoporphyrins

H. Nilsson[1], J. Johansson[1,2], K. Svanberg[1,3], S. Svanberg[1,2], G. Jori[4], E. Reddi[4], A. Segalla[4], D. Gust[5], A.L. Moore[5] & T.A. Moore[5]

[1]*Lund University Medical Laser Center and* [2]*Department of Physics, Lund Institute of Technology, PO Box 118, S-221 00 Lund, Sweden;* [3]*Department of Oncology, Lund University Hospital, S-221 85 Lund, Sweden;* [4]*Department of Biology, Padova University, Via Trieste 75, 351 21 Padova, Italy;* [5]*Department of Chemistry and Biochemistry, Center for the Study of Early Events in Photosynthesis, Arizona State University, Tempe, Arizona 85287-1604, USA.*

**Summary** Laser-induced fluorescence (LIF) was used to characterise the localisation of an intravenously administered trimethylated carotenoporphyrin [$CP(Me)_3$] and a trimethoxylated carotenoporphyrin [$CP(OMe)_3$] in an intramuscularly transplanted malignant tumour (MS-2 fibrosarcoma) and healthy muscle in female Balb/c mice, 3, 24, 48 and 96 h post injection. The fluorescence was induced with a dye laser pumped by a nitrogen laser, emitting light at 425 nm. The fluorescence spectra were recorded in the region 455-760 nm using a polychromator equipped with an image-intensified CCD camera. The tumour/peritumoral muscle ratio was about 5:1 for $CP(Me)_3$ and about 6:1 for $CP(OMe)_3$ in terms of the background-free fluorescence intensity, which peaked at about 655 nm. By including the endogenous tissue fluorescence, the contrast was further enhanced by a factor of approximately 2.

In some clinical situations, tumour diagnostic techniques may fail to localise precancerous lesions and superficial tumours, e.g. in the tracheobronchial area, the oesophagus, the urinary bladder and the ear/nose/throat region. Biopsy sampling is often performed in a blind way during conventional tumour screening procedures; hence, the malignant areas may not be localised. Therefore, there is a need to develop more sensitive methods. Laser-induced fluorescence (LIF) in conjunction with different photosensitisers is a new, rapidly evolving modality which has exhibited promising results in early tumour localisation (Cortese *et al.*, 1979; Doiron *et al.*, 1979). Most frequently, various derivatives of porphyrins, such as haematoporphyrin derivative (HpD) (commercial name Photofrin), have been used. These substances give rise to a characteristic red fluorescence when excited in the UV or near-UV region. As accumulation of the photosensitising substance occurs in premalignant and malignant areas, diseased tissue is characterised by a higher fluorescence intensity in the red wavelength region, compared with most non-diseased tissues. For example, LIF of tumours in the bronchial tree and the urinary bladder in conjunction with low-dose HpD in clinical diagnostics has been performed (Lam *et al.*, 1990; Baert *et al.*, 1992). Premalignant and *in situ* malignant lesions were localised.

Although HpD has tumour-localising properties with a discrimination towards normal tissue of about 2-3:1 for various malignant lesions, several factors limit its widespread use as a photodiagnostic agent, including the heterogeneous chemical composition and the presence of non-fluorescent aggregated material, the limited selectivity and the prolonged persistence of general cutaneous photosensitivity if administered in therapeutic doses (Dougherty, 1987; Razum *et al.*, 1987). The development of new sensitisers has mainly emphasised their therapeutic effects, including high single oxygen yield for the induction of tumour necrosis and light absorption at wavelengths close to 700 nm for deep light penetration. Also, less skin photosensitisation, as compared with HpD, is a major goal. Substances which have been studied include phthalocyanines (Pcs) (Spikes, 1986), various derivatives of cholorphyll, chlorins (Nelson *et al.*, 1987), purpurins (Kessel, 1989) and some additional porphyrin derivatives. Lately, $\delta$-aminolaevulinc acid (ALA) (Kennedy *et al.*, 1990: Kennedy & Pottier, 1992; Grant *et al.*, 1993; Svanberg *et al.*, 1994), a precursor to haem, and benzoporphyrin derivative (BPD) (Richter *et al.*, 1987, 1988, 1990) have been investigated. MACE (mono-aspartyl chlorin $e_6$) does not induce any cutaneous photosensitivity at all, because of a fast clearance rate (Nelson *et al.*, 1987).

Recently, a new class of tumour-localising substances has been developed solely for tumour demarcation capability, without any photosensitising effect. These substances consist of carotenoid polyenes covalently linked to porphyrins, in which the tetraaryl porphyrin moiety carries either three methyl (Me) or three methoxy (OMe) groups (Reddi *et al.*, 1994). These carotenoporphyrins (CPs) have the unique capability of mimicking carotenoid photoprotection such as occurs in the photosynthetic reaction centres of green plants. The carotene moiety rapidly quenches the porphyrin triplet state, thus preventing the porphyrin-sensitised formation of cytotoxic $^1O_2$ (Gust *et al.*, 1992*a*). Accordingly, CPs cannot be used for photodynamic therapy (PDT), but are valuable alternatives for LIF-mediated detection of malignant lesions, because they are designed to reduce damage to skin and other organs.

In this paper, we report on laser-induced fluorescence measurements in mice injected with $CP(Me)_3$ or $CP(OMe)_3$. The acquired spectra were used to characterise a transplanted fibrosarcoma and normal muscle (peritumoral tissue), following excitation at 425 nm. Similar studies have been carried out previously using other substances such as HpD (Svanberg *et al.*, 1986), haematoporphyrin (Hp) (Andersson-Engels *et al.*, 1989*a*), polyhaematoporphyrin ester (PHE) (Andersson-Engels *et al.*, 1989*a*), tetrasulphonated Pc (TSPc) (Andersson-Engels *et al.*, 1989*a*) and BPD-monoacid (BPD-MA) (Andersson-Engels *et al.*, 1993), in other experimental tumour systems.

In addition to simply using the substance-related fluorescence, the endogenous fluorescence emanating from natural fluorophores in the tissue may also be included in order to enhance the tumour demarcation towards healthy peritumoral tissue. It has been shown that the autofluorescence, with its intensity maximum at about 500 nm when excited in the near-UV region, shows a lower intensity in tumour than in normal tissue (Ankerst *et al.*, 1984; Profio & Sarnaik, 1984; Lohmann *et al.*, 1989; Andersson-Engels *et al.*, 1989*b*, 1991*a*; Cothren *et al.*, 1990; Hung *et al.*, 1991; Baert *et al.*, 1992). To some extent, the reason for the decreased autofluorescence seems to be a decrease in the amount of oxidised forms of flavins in malignant tissue

Correspondence: Dr. K. Svanberg, Department of Oncology, Lund University Hospital, S-221 85 Lund, Sweden.
Received 21 January 1994; and in revised form 9 June 1994.

(Hung *et al.*, 1991). Another reason for the decrease in tumour autofluorescence might be a change in the relative amounts of the highly fluorescent reduced form of nicotinamide adenine dinucleotide (NADH) and the weakly fluorescent oxidised form $NAD^+$ in malignant tumours (Svanberg *et al.*, 1986; Lohmann & Hugo, 1989; Andersson-Engels *et al.*, 1991*b*). Tumour-induced transformation of the tissue collagen, which normally exhibits a high fluorescence intensity at about 390 nm, may also be important. When tumour cells invade a host tissue, tumour-specific collagenase type IV is released (Poulsom *et al.*, 1992; Campo *et al.*, 1992), which may destroy the healthy collagen with loss of the high, collagen-specific fluorescence intensity.

Results from *in vivo* clinical tumour investigations with UV and near-UV excitation clearly show a decrease in the autofluorescence signal in the tumour compared with the peritumoral tissue (Hung *et al.*, 1991; Baert *et al.*, 1992). By measuring the ratio between the exogenous fluorescence, which increases in tumour, and the autofluorescence, which decreases, the tumour/peritumoral tissue demarcation is enhanced. In addition, the designation of a dimensionless ratio also implies an insensitivity to fluctuations in excitation and detection equipment and variations in surface topography or differences in the distance between the optical fibre and the tissue being analysed.

### Materials and methods

### *Chemicals*

The carotenoporphyrins were synthesised at Arizona State University according to the procedures previously described (Gust *et al.*, 1992*b*). Their structural formulas are shown in Reddi *et al.* (1994). A 5 mg aliquot of carotenoporphyrin was added to 0.5 ml of Cremophor and sonicated until the CP was completely dispersed in the emulsifier agent. The suspension was added to 0.15 ml of absolute ethanol, sonicated again and taken to a final volume of 10 ml by the stepwise addition of a saline solution at pH 7.4. The final Cremophor emulsion was filtered through a 0.45 μm filter and the CP concentration (0.45–0.5 mg) was determined spectrophotometrically after a 1:200 dilution of a small aliquot of emulsion in absolute ethanol and using an extinction coefficient of $3.74 \times 10^5$ $mmol^{-1}$ $cm^{-1}$ at 420 nm. The CPs in the Cremophor emulsion were i.v. injected to tumour-bearing mice at a dose of 4.2 μmol $kg^{-1}$ [5.0 and 5.2 mg $kg^{-1}$ of $CP(Me)_3$ and $CP(OMe)_3$ respectively]. Cremophor EL was supplied by Sigma. All other chemicals and solvents were analytical-grade reagents.

### *Animals and tumour*

Female Balb/c mice (18–22 g body weight) received 0.2 ml of a cell suspension containing $10^6$ MS-2 fibrosarcoma cells $ml^{-1}$. The tumour grows intramuscularly in the right hind leg 6 days before the first day of measurement (Reddi *et al.*, 1994).

### In vivo *and* ex vivo *fluorescence measurements*

Figure 2 illustrates the method used for the fluorescence measurements. A scan over healthy muscle and tumour tissue was performed, and also, in the *ex vivo* situation, a reference measurement in the non-diseased left hind legs of the mice was taken. The *in vivo* scans were performed after an i.p. injection of approximately 0.09 ml Ketalar (50 mg $ml^{-1}$) from Parke Davis, Milan. After the animal was anaesthetised, the skin over the tumour and surrounding mucscle was cut open, the scan was performed and the animal was sacrificed with ether. In the dead mice, the optical fibre was inserted a few millimetres into the tissue, where fluorescence measurements were carried out.

Two points were measured at 5 and 2 mm outside the tumour in healthy muscle. Inside the tumour, five points were chosen for measurements, the first and last point being about 1 mm from the tumour border with healthy muscle, and the other points being equally spaced along a line bisecting the tumour. The diameters of the tumours were about 15 mm at the time of analysis. The tumour model used (fibrosarcoma MS-2) was not optimal in that necrotic areas appeared rather quickly. No major necrosis was present in the measurements 3 and 24 h post injection, and the few points where recordings had been taken from necrotic areas were deleted before the data analysis was performed. In the case of the 48 h measurements, the amount of necrosis had increased somewhat, but the same procedure was followed. For the 96 h groups, no points whatsoever were deleted, which is reflected in the great standard deviations of the fluorescence signal. This is especially true for the substance $CP(Me)_3$, which, at 96 h, exhibited huge standard deviations as a consequence of very necrotic tumours. The necrosis also predisposed to haemorrhages, which further interfered with the fluorescence because of light reabsorption by blood in the green–yellow spectral region.

### *Equipment*

The optical set-up used for the recordings of laser-induced fluorescence is similar to one previously described (Andersson-Engels *et al.*, 1989*a*, 1991*a*) and will only be briefly described here. The excitation source was a nitrogen laser (Laser Science VSL-337ND) used alone or as pump source for a compact dye laser (Laser Science DLM220). The output pulse energy of the nitrogen laser was 180 μJ, the pulse duration 3 ns and the repetition rate about 10 Hz. For the dye laser the output energy was up to 20 μJ and the wavelength could be varied by turning a grating. For these measurements, the wavelength was set at 425 nm. The laser light was focused onto the tip of a fluorescence-free 600 μm optical fibre and the distal end of the fibre was held in contact with the animal tissue. The fluorescence guided back through the same fibre was transmitted through a dichroic mirror, which at the same time blocks out the reflected excitation light, and focused at the 100 μm entrance slit of a polychromator (Acton SP-275). A cut-off filter at 455 nm was used to remove any residual elastically scattered excitation light. The detector was a 576 × 384 pixel image-intensified charge-coupled device (CCD) camera (Spectroscopy Instr. ICCD-576G/R) cooled to −20°C. In total, about 2,500 fluorescence spectra were recorded and stored on floppy discs for evaluation. The fluorescence from 50 laser pulses was integrated for each spectrum, although in most cases useful signal-to-noise was obtained in single shot.

### Results and Discussion

Figure 1 shows six fluorescence spectra recorded *in vivo* from the exterior part of the tumours and from healthy muscle fasciae, surrounding the malignant tumours, in two animals injected 3 h earlier with (a) $CP(Me)_3$ and (b) $CP(OMe)_3$, and (c) in a non-injected control animal. The heavy line denotes malignant tissue and the thin one healthy muscle.

The carotenoporphyrin-specific fluorescence is characterised by two peaks. In the case of $CP(Me)_3$ the peaks are at 654 and 720 nm, whereas they are slightly shifted to the red in the case of $CP(OMe)_3$, appearing at 658 and 722 nm. The fluorescence intensities at the peaks were evaluated with the background fluorescence subtracted, as marked in the Figure. The first substance-specific free-standing peak is denoted 'A' and the endogenous fluorescence 'B'. The ratio between A and the second substance-related peak varied slightly according to substance injected and time elapsed since injection. The variations, however, were insignificant and the ratios were about 2.1 for $CP(Me)_3$ and 2.3 for $CP(OMe)_3$. The values did not differ significantly between tumour and normal muscle. The tissue autofluorescence has its maximum at about 510 nm, for the excitation wavelength chosen, but was

**Figure 1** *In vivo* spectra taken 3 h post i.v. injection of 4.2 μmol $kg^{-1}$ of **a**, $CP(Me)_3$ and **b**, $CP(OMe)_3$. Below, in **c**, two spectra are shown from a non-injected control animal. These *in vivo* measurements were carried out as exterior scans over healthy, peritumoral muscle fascia and tumour capsule, with the skin removed. The thin line represents a location outside the tumour, on healthy muscle fascia, and the heavy line represents a location inside the tumour. 'A', at the top of the figure, denotes the background-free peak, located at 654 nm in the case of $CP(Me)_3$, and 'B' represents the autofluorescence, evaluated at 490 nm.

evaluated at 490 nm (B) – a wavelength frequently used for monitoring endogenous fluorescence.

The spectra from the injected animals show, when compared with those from the control animal, that the carotenoporphyrins are retained with good selectivity in tumour tissue, as compared with surrounding muscle, in the *in vivo* situation. We found that on average for all groups of mice $CP(Me)_3$ showed about five times higher fluorescence in tumour than in muscle, and that the corresponding value was about 6 for $CP(OMe)_3$, according to measurements based on the substance-related peak A. The autofluorescence in the blue–green wavelength region (B) has a lower intensity in tumour tissue than in the surrounding muscle, although the excitation wavelength used (425 nm) is not the optimal one for exciting NADH and collagen.

Figure 2 shows a typical fluorescence scan, recorded *ex vivo*, with the fibre inserted into the tissue in an animal injected with (a) $CP(Me)_3$ or (b) $CP(OMe)_3$ 3 h earlier. The dual-peaked substance-related fluorescence is clearly seen in the points recorded from the tumour, including the border zone, while the muscle tissue exhibits much lower fluorescence in the red wavelength region. The autofluorescence also shows a lower intensity in the tumour tissue than in the muscle in this *ex vivo* scan. By comparing the spectra in Figures 1 and 2, it can be concluded that no major differences exist in the shape or relation of the various peaks and evaluation points in the spectra, whether the spectra were recorded exteriorly, *in vivo*, or interiorly, *ex vivo*. The higher fluorescence values in Figure 2b for $CP(OMe)_3$ could perhaps be attributed to the greater build-up of the substance $CP(OMe)_3$ than of $CP(Me)_3$ in tumour, as discussed in Reddi *et al.* (1994).

In several spectra, as seen in some of the graphs in Figure 2, a small peak at approximately 590 nm is observed. The same peak was also seen in the non-injected control animals. A similar peak has been observed also in clinical fluorescence measurements, e.g. in astrocytomas and human tonsil cancers (Andersson-Engels *et al.*, 1991*a*; Strömblad *et al.*, submitted). It is probably attributable to some endogenous porphyrin, perhaps bacterially synthesised (Harris & Werkhaven, 1987). Another possibility is that the fluorescence is related to some metalloporphyrin, probably zinc haematoporphyrin (ZnHp), which is synthesised by the tumour itself (Moan, 1986; Plus, 1992). No significant difference between tumour and normal tissue could be seen in the fluorescence intensity at this wavelength, in the measurements carried out 3 and 24 h post injection. This is true for both the injected and the non-injected control animals, evaluated at corresponding times. The intensities were in the same range for the control animals and the animals evaluated 3 h after administration of the two carotenoporphyrins, which all had had their tumours transplanted at the same time. In the measurements 48 and 96 h post administration, with much necrosis, the tumours exhibited somewhat higher intensities at 590 nm, but with no great significance, owing to large fluctuations in the signal.

The evaluated data from all the tumour scans are shown in Figure 3a for $CP(Me)_3$ and Figure 3b for $CP(OMe)_3$, with the *in vivo* externally recorded scans at the top and the *ex vivo* internally measured scans below, in each figure. Each graph represents an average of the measurements for each group of animals, usually consisting of five mice, but in two cases [$CP(Me)_3$ and $CP(OMe)_3$ at 48 h] only of four, evaluated at 3, 24, 48 or 96 h post administration. Both the exterior and interior scans are shown in terms of the averaged background-free substance-related fluorescence intensity, A, expressed in units relative to a fluorescence standard and as the ratio between A and the tissue autofluorescence B. In all graphs, the vertical bars indicate $\pm$ 1 standard deviation. The general trend for $CP(Me)_3$ is that the standard deviations increase with time after injection. This phenomenon might partly be caused by a greater amount of necrosis in the later measurements, which results in larger fluctuations in the fluorescence signal.

Figure 3 clearly shows that the main fluorescence peak (A) has a larger intensity in tumour for both substances. On average, the intensity increase in tumour compared with muscle is about 5 for $CP(Me)_3$ and 5.6–6.8 for $CP(OMe)_3$. An important property of both substances is the tumour selectivity demonstrated in the scans with the registration 2 mm outside the tumour, in muscle, where the intensity is approximately equal to the point 5 mm outside the tumour; i.e. no increase is seen on approaching the tumour. The fluorescence intensity is not elevated until the tumour is reached. Except in the case of the data 48 and 96 h post injection with $CP(Me)_3$, the standard deviations are small. The intensity of the carotenoporphyrin-related fluorescence (A) increases over time up to 48 h for $CP(Me)_3$ and then starts falling off, probably reflecting an initial build-up of the substance in the tissues and then a consecutive clearing. In the case of $CP(OMe)_3$, A decreases slightly when going from 3 h to 24 h post injection, then it remains relatively constant up to 48 h and, finally, falls off during the following 48 h. (Note the change in scales with increasing times.) This is probably because the substance is cleared from the tissues. The tumour/muscle ratio, in terms of the free-standing peak A is, for $CP(Me)_3$, 5.5 at 3 h, 5.9 at 24 h, 7.5 at 48 h and 3.4 at 96 h. The corresponding ratio for $CP(OMe)_3$ is 3.8, 5.4, 9.1 and 8.4. In the case of $CP(Me)_3$, the tumour/muscle ratio remains relatively constant over time, since the fluorescence intensities decrease in the same proportions in muscle and in tumour. For $CP(OMe)_3$, the tumour/muscle ratio increases at 48 and 96 h post administration, indicating that the substance is retained for a longer time in tumour than in healthy muscle.

If the endogenous fluorescence is included, forming the ratio A/B, the tumour/muscle ratio is enhanced to 7.3 at 3 h, 9.5 at 24 h, 12.5 at 48 h and 7.9 at 96 h, and 8.2 at 3 h, 9.3 at 24 h, 10.3 at 48 h and 11.2 at 96 h for $CP(Me)_3$ and $CP(OMe)_3$ respectively. The increase in the tumour/muscle ratio varies between 27% and 400%, with an average of about 95%, when the autofluorescence is included. The extremely high values of several hundred per cent contrast enhancement probably are due to a reduction in the autofluorescence because of necrosis, as pointed out previously, which also means that the contrast is enhanced at the expense of a greater uncertainty in the results (see, for

**Figure 2** Laser-induced fluorescence spectra recorded *ex vivo* 3 h post i.v. injection with **a**, 5.0 mg $kg^{-1}$ b.w. $CP(Me)_3$ and **b**, 5.2 mg $kg^{-1}$ b.w. $CP(OMe)_3$. The spectra were recorded in 20-mm-long scans in peritumoral and tumoral tissue in the tumour leg and as a single point in healthy muscle in the other leg. The measurements were carried out with the fibre probe interstitially located in the tissues. The fluorescence intensity is expressed in relative units. Note that the scales are different in the two scans.

example Figure 3a, 96 h post injection *in vivo*). The main advantage of forming the ratio is that it is dimensionless, which means that the results are rather insensitive to fluctuations in the excitation source and the detection equipment, as well as to topographic and distance differences in the measurement procedure.

The results from Figure 3 are summarised in Figure 4. The tumour/peritumoral muscle ratio is plotted for the two carotenoporphyrins as a function of time after administration. Figure 4a shows the tumour/muscle ratio using the substance-related peak, A, as an average of all the *in vivo* and *ex vivo* measurements, whereas Figure 4b shows the same for

**Figure 3** Averages of the fluorescence intensity at the main peak 'A' (relative units) and the fluorescence ratio (A/B) in healthy muscle and tumour tissue in the different groups, as measured at 3, 24, 48 and 96 h post i.v. injection with **a**, 5.0 mg kg$^{-1}$ b.w. CP(Me)$_3$ and **b**, 5.2 mg kg$^{-1}$ b.w. CP(OMe)$_3$. (Note the change in scales with increasing times.) Five animals were analysed in each group, except for the two batches at 48 h, consisting of four animals each.

**Figure 4** Tumour demarcation (tumour/muscle ratio) at 3, 24, 48 and 96 h post i.v. injection with 4.2 μmol kg$^{-1}$ of CP(Me)$_3$ or CP(OMe)$_3$. Each graph displays the exterior (□) and interior (▲) measurement series. The tumour/muscle ratio was calculated using **a**, the background-free substance-related fluorescence intensity 'A' and **b**, the ratio of the fluorescence intensity 'A' and the endogenous fluorescence 'B'.

the ratio A/B. From Figure 3 and 4a it can be concluded that CP(OMe)$_3$ is cleared faster from muscle tissue than from tumour tissue, as compared with CP(Me)$_3$. When the native fluorescence is taken into account, the *in vivo* (exterior) and *ex vivo* (interior) measurements differ in demarcation power (i.e. tumour/muscle ratio) for both substances, mainly reflecting a large uncertainty in B. As already mentioned, the excitation wavelength used at 425 nm is not optimal for exciting the native fluorescence owing to the strong interference of blood absorption, which especially affects the interior measurements. Thus, the average of the interior demarcations is about 25–31% lower than the exterior ones.

In order to assess the actual consistency and reliability in the demarcation between diseased and healthy tissue, a discrimination function ($D_k$) was defined as follows (Andersson-Engels *et al.*, 1991*a*):

$$D_k = \frac{\text{Average}_{\text{tumour}} - \text{Average}_{\text{muscle}}}{\sqrt{(\sigma_{\text{tumour}}^2 + \sigma_{\text{muscle}}^2)}}$$

where the averages denote the mean values of the fluorescence intensities at the various times after injection of the two carotenoporphyrins and at the different measurement locations (inside or outside the tumour regions). The value of $\sigma$ denotes one standard deviation. This function is a parallel to the '*z*-test', which means that it is a measure of how far apart the error bars of the two compared functions (in this case fluorescence intensity inside and outside tumour) are separated. In other words, the larger the $D_k$ value is, the more certain the demarcation. For these measurements, the number of points considered in each group and site (exterior or interior scan) was approximately 25–40.

Calculating the number of degrees of freedom (number of points − 2) and asuming a significance level of 5% ($P<0.05$), it can be found in tables that if ($D_k > 1.7$, the demarcation is significant to the aforementioned level. For the ratio A/B, this criterion was fulfilled for both the substances at 3 and 24 h post injection, whereas only CP(OMe)$_3$ fully passed the test for the later scans. Probably because of problems with the tumour model used, as previously explained, CP(Me)$_3$ did not show a sufficient $D_k$ in the later, exterior scans. For most groups, the values were exceeding 3, which indicates that the discrimination is excellent. When creating the discrimination coefficient for the peak A alone, about the same trend as for the ratio is followed, but, generally, with smaller values.

In conclusion, CP(Me)$_3$ and CP(OMe)$_3$ are shown to demarcate malignant tissue from normal, surrounding tissue at ratios of about 5:1 and 6:1, respectively, in the tumour model used. If the tissue autofluorescence is included, the demarcation is enhanced to 8–10:1 and 9–12:1, respectively, with a higher demarcation in the exterior scans. The carotenoporphyrins are quickly taken up by the kind of tumour used, and already show a good and consistent demarcation 3 h after administration, which is a good indication for possible future clinical use. CP(Me)$_3$ exhibited smaller standard deviations 3 h post injection; thus, this substance might be the one of choice in a clinical situation. However, at the injected doses, CP(OMe)$_3$ exhibits a more intense fluorescence at the main peak, A, than CP(Me)$_3$, which might indicate the possibility of a dose reduction in the former case. This could be of value in a clinical situation, because of the inherent substance characteristic of accumulating in the reticuloendothelial system, as described by Reddi *et al.* (1994).

This work was supported by the Swedish Cancer Society, the Swedish Board for Technical and Industrial Development and the Swedish Research Council of Engineering Sciences.

### References

ANDERSSON-ENGELS, S., ANKERST, J., JOHANSSON, J., SVANBERG, K. & SVANBERG, S. (1989*a*). Tumour marking properties of different haematoporphyrins and tetrasulphonated. phthalocyanine - a comparison. *Lasers Med. Sci.*, **4**, 115-123.

ANDERSSON-ENGELS, S., BRUN, A., KJELLÉN, E., SALFORD, L.G., STRÖMBLAD, L.-G., SVANBERG, K. & SVANBERG, S. (1989*b*). Identification of brain tumours in rats using laser-induced fluorescence and hematoporphyrin derivative. *Lasers Med. Sci.*, **4**, 241-249.

ANDERSSON-ENGELS, S., ELNER, Å., JOHANSSON, J., KARLSSON, S.-E., SALFORD, G., STRÖMBLAD, L.-G., SVANBERG, K. & SVANBERG, S. (1991*a*). Clinical recording of laser-induced fluorescence spectra for evaluation of tumour demarcation feasibility in selected clinical specialities. *Lasers Med. Sci.*, **6**, 415-424.

ANDERSSON-ENGELS, S., JOHANSSON, J., SVANBERG, K. & SVANBERG, S. (1991*b*). Fluorescence imaging and point measurements of tissue: applications to the demarcation of malignant tumours and atherosclerotic lesions from normal tissue. *Photochem. Photobiol.*, **53**, 807-814.

ANDERSSON-ENGELS, S., ANKERST, J., JOHANSSON, J., SVANBERG, K. & SVANBERG, S. (1993). Laser-induced fluorescence in malignant and normal tissue of rats injected with benzoporphyrin derivative. *Photochem. Photobiol.*, **57**, 978-983.

ANKERST, J., MONTÁN, S., SVANBERG, K. & SVANBERG, S. (1984). Laser-induced fluorescence studies of hematoporphyrin derivative (HpD) in normal and tumour tissue of rat. *Appl. Spectrosc.*, **38**, 890-896.

BAERT, L., BERG, R., VAN DAMME, B., D'HALLEWIN, M.A., JOHANSSON, J., SVANBERG, K. & SVANBERG, S. (1992). Clinical fluorescence diagnosis of human bladder carcinoma following low dose Photofrin injection. *Urology*, **41**, 322-330.

CAMPO, E., MERINO, M.J., LIOTTA, L., NEUMANN, R. & STETLER-STEVENSON, W. (1992). Distribution of the 72-kd type IV collagenase in nonneoplastic and neoplastic thyroid tissue. *Hum. Pathol.*, **23**, 1395-1401.

CORTESE, D.A., KINSEY, J.H., WOOLNER, L.B., SPENCER PAYNE, W., SANDERSON, D.R. & FONTANA, R.S. (1979). Clinical application of a new endoscopic technique for detection of *in situ* bronchial carcinoma. *Mayo Clin. Proc.*, **54**, 635-642.

COTHREN, R.M., RICHARDS-KORTUM, R., SIVAC, Jr, M.V., FITZMAURICE, M., RAVA, R.P., BOYCE, G.A., DOXTADER, M., BLACKMAN, R., IVANE, T.B., HAYES, G.B., FELD, M.S. & PETRAS, R.E. (1990). Gastrointestinal tissue diagnosis by laser-induced fluorescence spectroscopy at endoscopy. *Gastrointest. Endosc.*, **36**, 105-111.

DOIRON, D.R., PROFIO, E., VINCENT, R.G. & DOUGHERTY, T.J. (1979). Fluorescence bronchoscopy for detection of lung cancer. *Chest*, **76**, 27-32.

DOUGHERTY, T.J. (1987). Studies on the structure of porphyrins contained in Photofrin® II. *Photochem. Photobiol.*, **46**, 569-573.

GRANT, W.E., HOPPER, C., MACROBERT, A.J., SPEIGHT, P.M. & BROWN, S.G. (1993). Photodynamic therapy of oral cancer: photosensitisation with systemic aminolaevulinic acid. *Lancet*, **342**, 147-148.

GUST, D., MOORE, T.A., MOORE, A.L., DEVADOSS, C., LIDDELL, P.A., HERMANT, R., NIEMAN, R.A., DEMANCHE, L.J., DEGRAZIANO, J.M. & GOUNI, I. (1992*a*). Triplet and singlet energy transfer in carotene-porphyrin dyads: role of the linkage bonds. *J. Am. Chem. Soc.*, **114**, 3590-3603.

GUST, D., MOORE, T.A., MOORE, A.L. & LIDDELL, P.A. (1992*b*). Synthesis of carotenoporphyrin models for photosynthetic energy and electron transfer. In *Methods in Enzymology* Vol. 213, Packer, L. (ed.) pp. 87-100. Academic Press: San Diego.

HARRIS, D.M. & WERKHAVEN, J. (1987). Endogenous porphyrin fluorescence in tumors. *Lasers Surg. Med.*, **7**, 467-472.

HUNG, J., LAM, S., LERICHE, J.C. & PALCIC, B. (1991). Autofluorescence of normal and malignant bronchial tissue. *Lasers Surg. Med.*, **11**, 99-105.

KENNEDY, J.C. & POTTIER, R.H. (1992). Endogenous protporphyrin IX, a clinically useful photosensitizer for photodynamic therapy. *J. Photochem. Photobiol.*, **14**, 275-292.

KENNEDY, J.C., POTTIER, R.H. & PROSS, D.C. (1990). Photodynamic therapy with endogenous protoporphyrin IX: Basic principles and present clinical experience. *J. Photochem. Photobiol.*, **6**, 143-148.

KESSEL, D. (1989). Determinants of photosensitization by purpurins. *Photochem. Photobiol.*, **50**, 169-174.

LAM, S., PALCIC, B., McLEAN, D., HUNG, J., KORBERLIK, M. & PROFIO, A.E. (1990). Detection of early lung cancer using low dose Photofrin II. *Chest*, **97**, 333-337.

LOHMANN, W. & HUGO, F. (1989). The effect of NADH on different human and mouse cell lines. *Naturwissenschaften*, **76**, 72-74.

LOHMANN, W., MUSSMANN, J., LOHMANN, C. & KÜNZEL, W. (1989). Native fluorescence of unstained cryo-sections of the cervix uteri compared with histological observations. *Naturwissenschaften*, **76**, 125-127.

MOAN, J. (1986). Yearly review: porphyrin photosensitization and phototherapy. *Photochem. Photobiol.*, **43**, 681-690.

NELSON, J.S., ROBERTS, W.G. & BERNS, M.W. (1987). *In vivo* studies on the utilization of mono-L-aspartyl chlorin (NPe6) for photodynamic therapy. *Cancer Res.*, **47**, 4681-4685.

PLUS, R. (1992). A review of *in vivo* studies of porphyrins and unexpected fluorescences. An interpretation of the results. *Med. Hypotheses*, **37**, 49-57.

POULSOM, R., PIGNATELLI, M., STETLER-STEVENSON, W.G., LIOTTA, L.A., WRIGHT, P.A., JEFFERY, R.E., LONGCROFT, J.A., ROGERS, L. & STAMP, G.W.H. (1992). Stromal expression of 72 kda type IV collagenase (MMP-2) and TIMP-2 mRNAs in colorectal neoplasia. *Am. J. Pathol.*, **141**, 389-396.

PROFIO, A.E. & SARNAIK, J. (1984). Fluorescence of HpD for tumor detection and dosimetry in photoradiation therapy. In *Porphyrin Localization and Treatment of Tumors*, Doiron, D.R. & Gomer, C.J. (eds) pp. 163-175. Alan R. Liss: New York.

RAZUM, N., BALCHUM, O.J., PROFIO, A.E. & CARSTENS, F. (1987). Skin photosensitivity; duration and intensity following intravenous HpD and DHE. *Photochem. Photobiol.*, **46**, 925-928.

REDDI, E., SEGALLA, A., JORI, G., KERRIGAN, P.K., LIDDELL, P.A., MOORE, A.L., MOORE, T.A. & GUST, D. (1994). Carotenoporphyrins as selective photodiagnostic agents for tumours. *Br. J. Cancer*, **69**, 40-45.

RICHTER, A.M., KELLY, B., CHOW, J., LIU, D.J., NEIL TOWERS, G.H., DOLPHIN, D. & LEVY, J.G. (1987). Preliminary studies on a more effective phototoxic agent than hematoporphyrin. *J. Natl Cancer Inst.*, **79**, 1327-1332.

RICHTER, A.M., STERNBERG, E.D., WATERFIELD, E., DOLPHIN, D. & LEVY, J.G. (1988). Characterization of benzoporphyrin derivative, a new photosensitizer. *SPIE*, **997**, 132-138.

RICHTER, A.M., CERRUTI-SOLA, S., STERNBERG, E.D., DOLPHIN, D. & LEVY, J.G. (1990). Biodistribution of tritiated benzoporphyrin derivative ($^3$H-BPD-MA), a new potent photosensitizer, in normal and tumor-bearing mice. *J. Photochem. Photobiol.*, **5**, 231-244.

SPIKES, J.D. (1986). Phthalocyanines as photosensitizers in biological systems and for the photodynamic therapy of tumors. *Photochem. Photobiol.*, **43**, 691-699.

STRÖMBLAD, L.-G., SALFORD, L., ANDERSSON-ENGELS, S., BRUN, A., JOHANSSON, J. & SVANBERG, K. Peroperative recording of laser-induced fluorescence from human and rodent malignant astrocytomas. *J. Neurosurg* (submitted).

SVANBERG, K., KJELLÉN, E., ANKERST, J., MONTÁN, S., SJÖHOLM, E. & SVANBERG, S. (1986). Fluorescence studies of hematoporphyrin derivative in normal and malignant rat tissue. *Cancer Res.*, **46**, 3803-3808.

SVANBERG, K., ANDERSSON, T., KILLANDER, D., WANG, I., STENRAM, U., ANDERSSON-ENGELS, S., BERG, R., JOHANSSON, J. & SVANBERG, S. (1994). Photodynamic therapy of non-melanoma malignant tumours of the skin utilizing topical δ-amino levulinic acid sensitization and laser irradiation. *Br. J. Dermatol.*, **130**, 743-751.


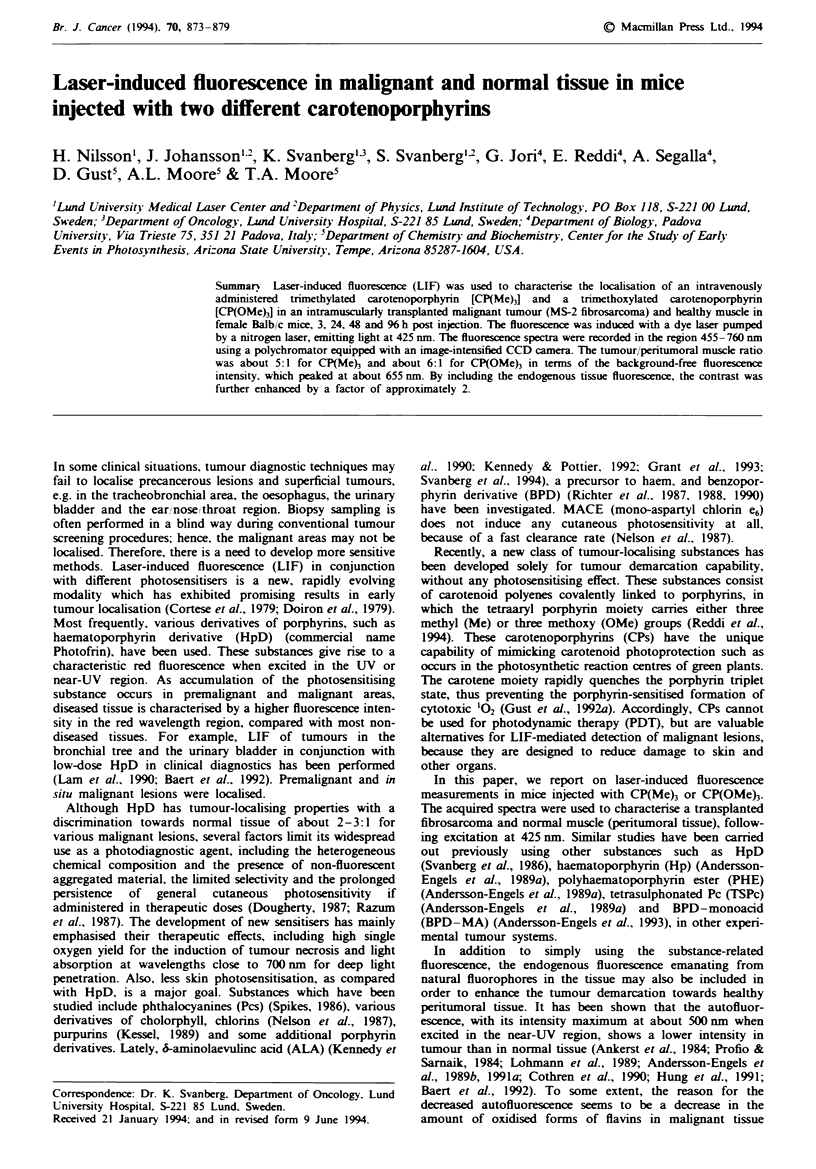

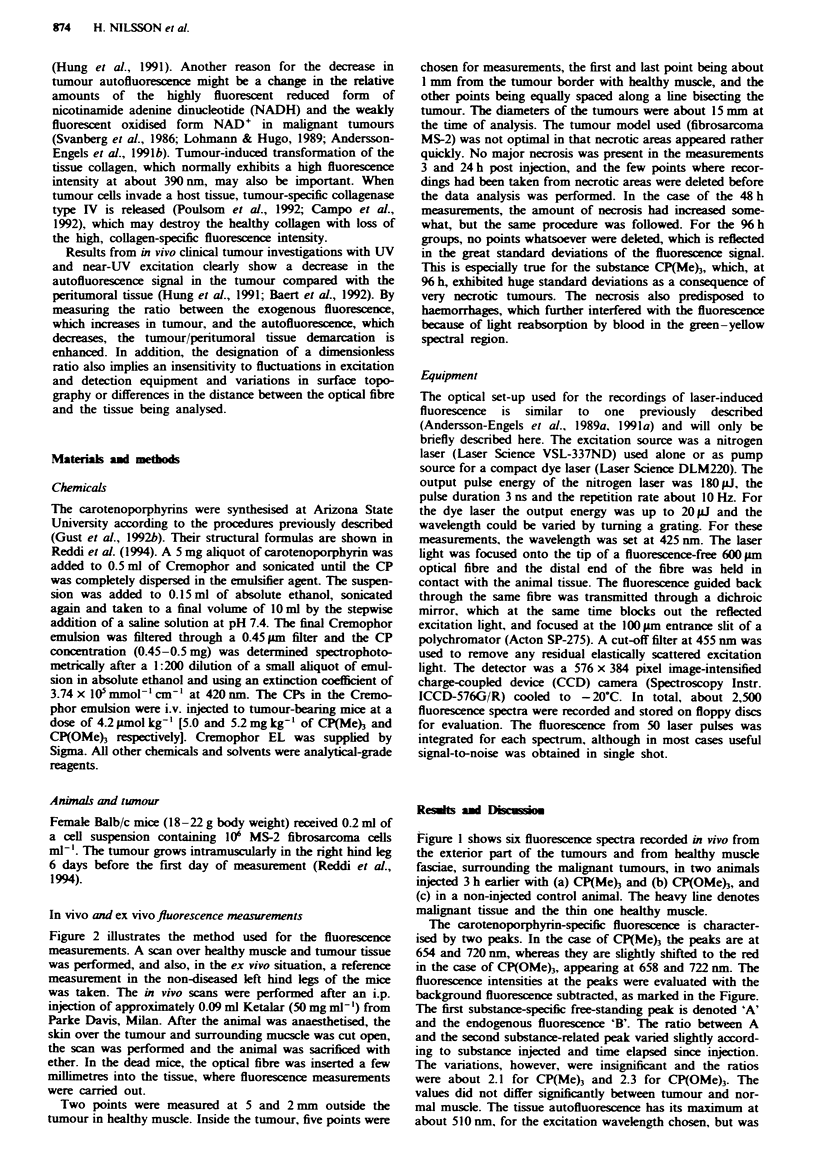

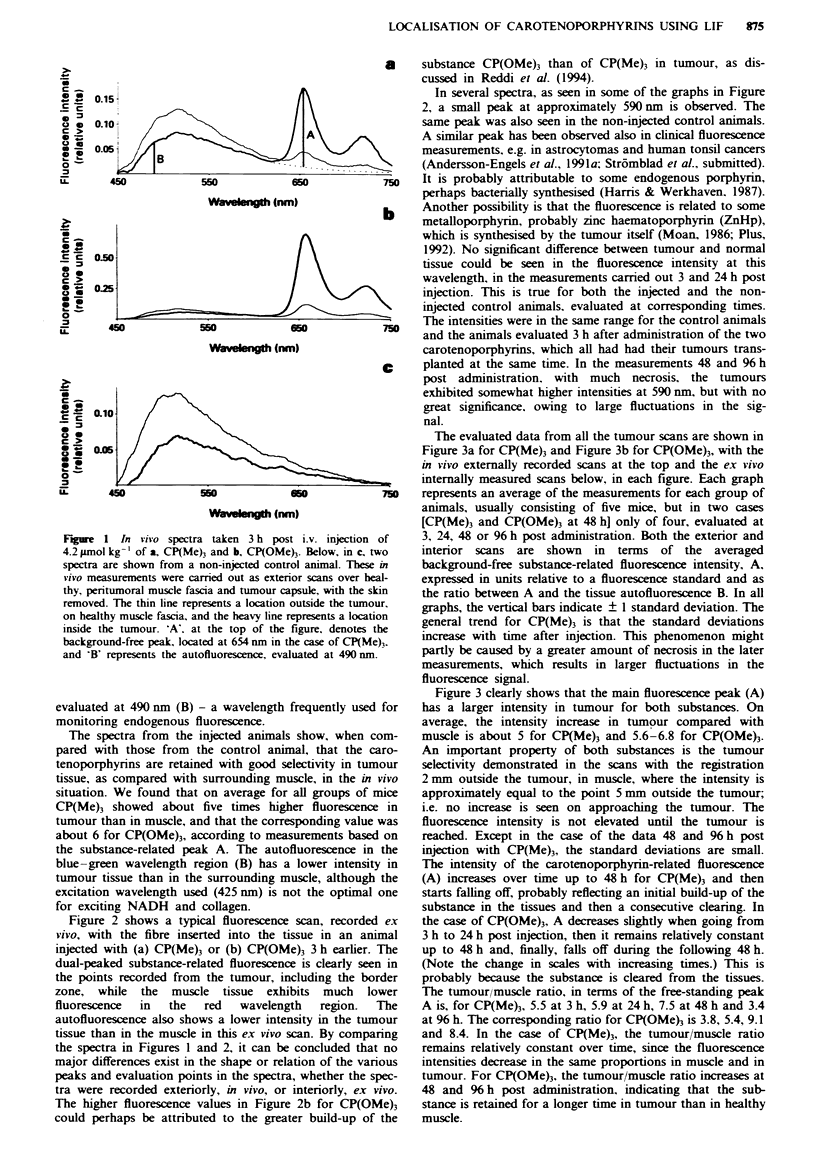

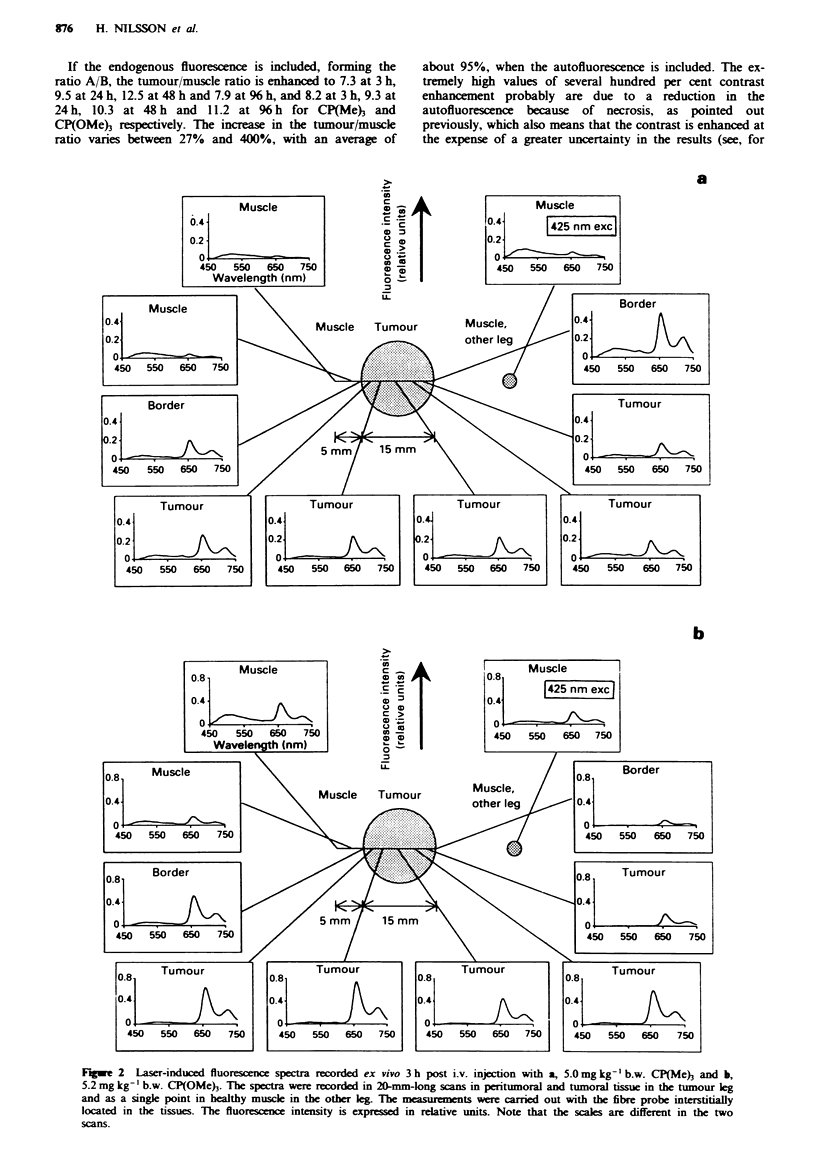

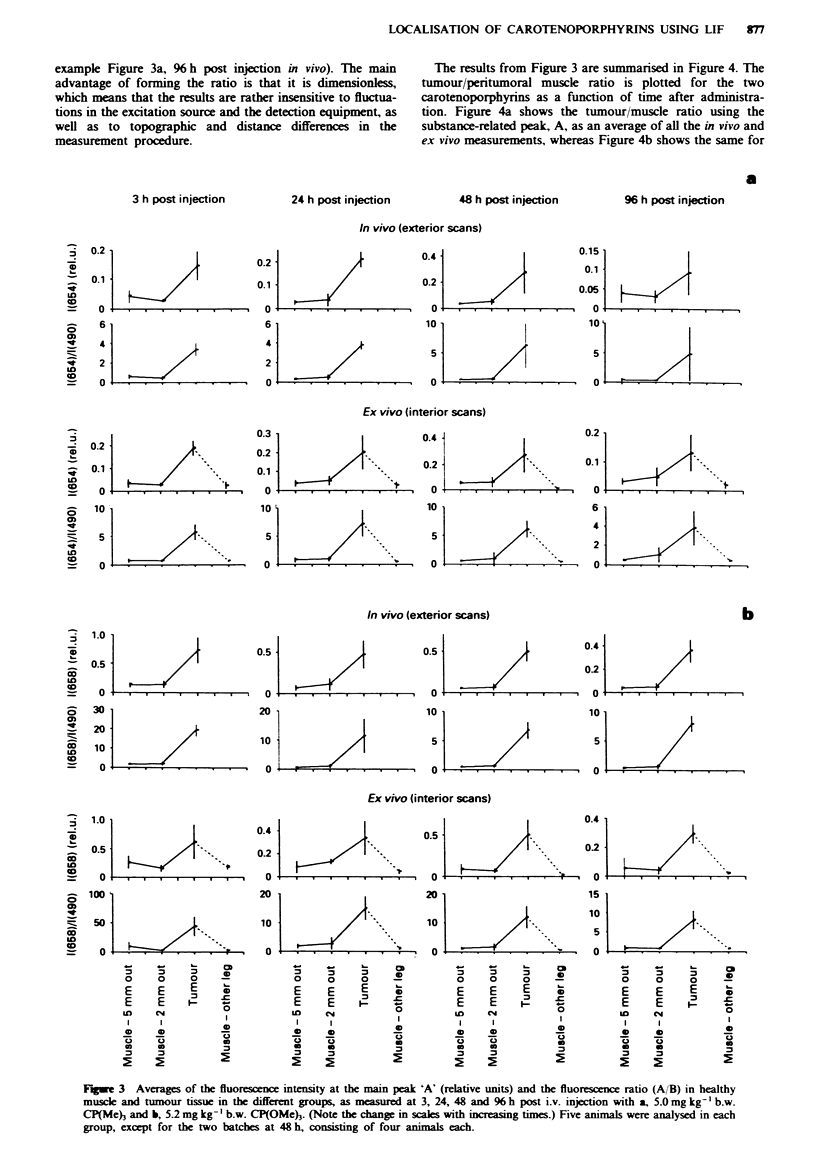

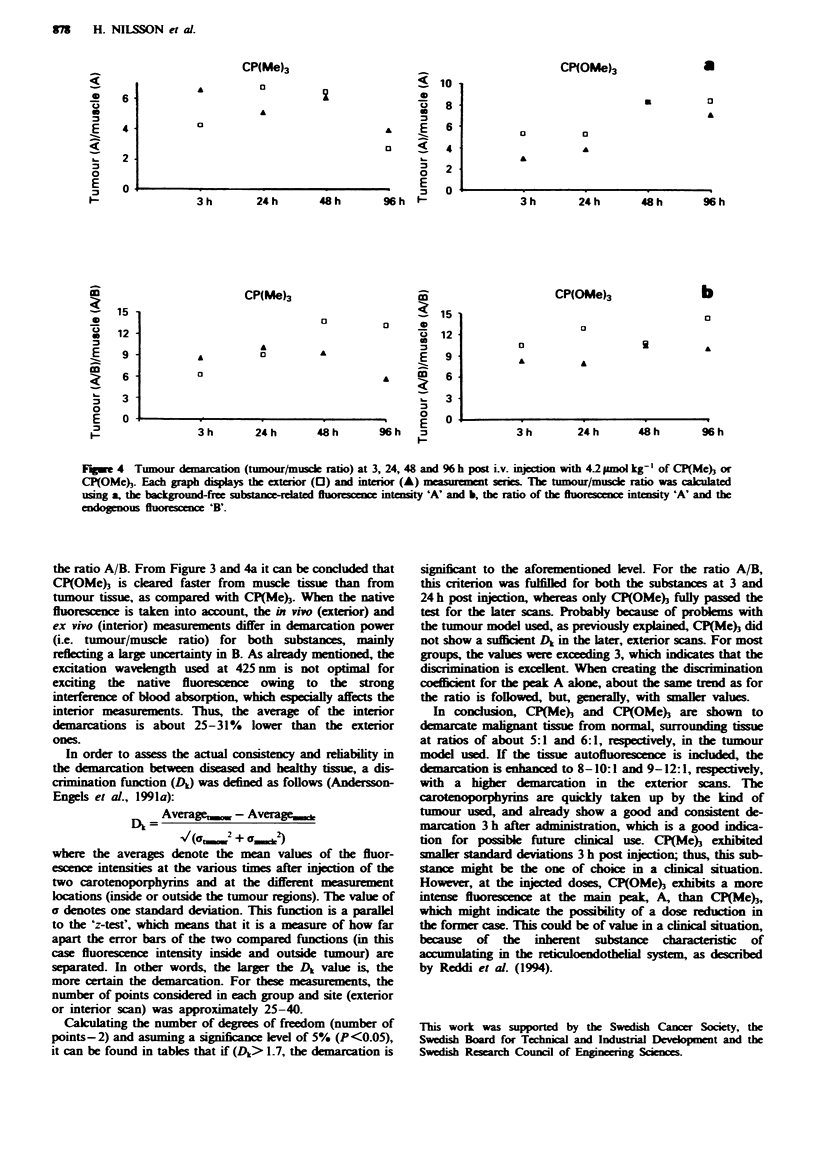

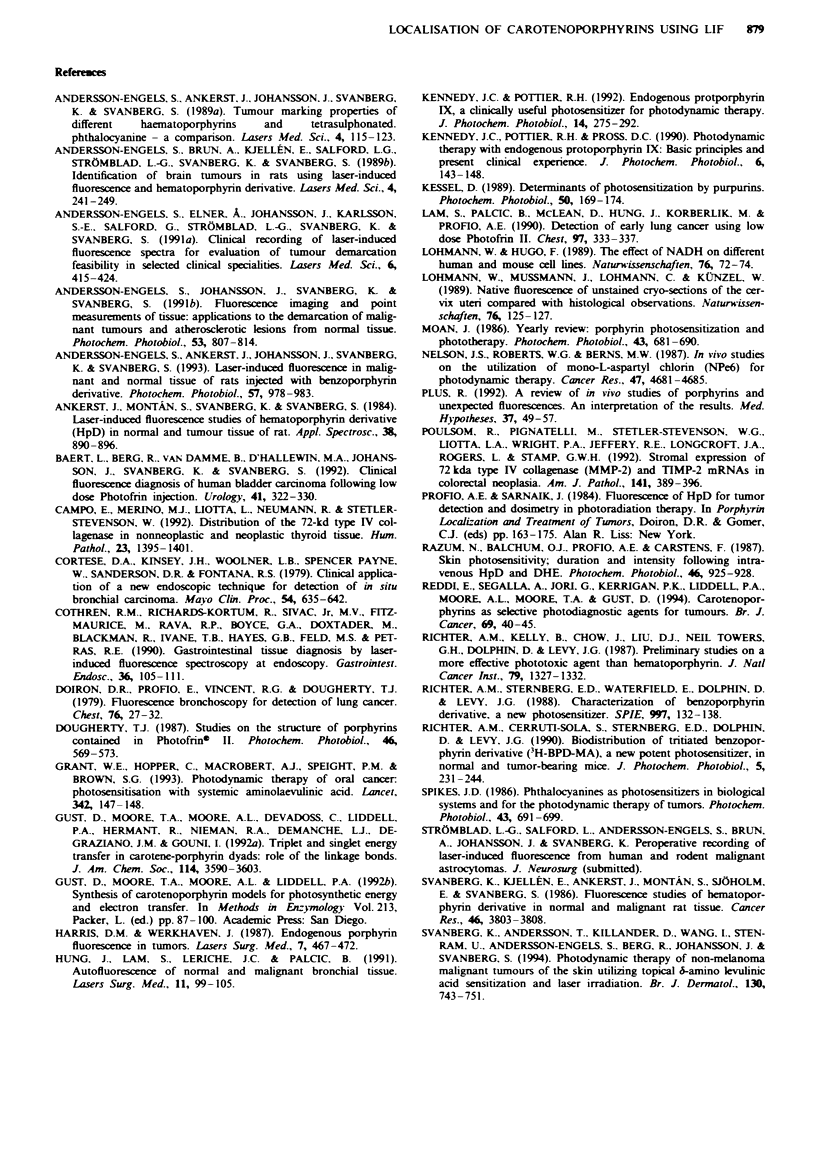


## References

[OCR_00825] Andersson-Engels S., Ankerst J., Johansson J., Svanberg K., Svanberg S. (1993). Laser-induced fluorescence in malignant and normal tissue of rats injected with benzoporphyrin derivative.. Photochem Photobiol.

[OCR_00816] Andersson-Engels S., Johansson J., Svanberg K., Svanberg S. (1991). Fluorescence imaging and point measurements of tissue: applications to the demarcation of malignant tumors and atherosclerotic lesions from normal tissue.. Photochem Photobiol.

[OCR_00835] Baert L., Berg R., Van Damme B., D'Hallewin M. A., Johansson J., Svanberg K., Svanberg S. (1993). Clinical fluorescence diagnosis of human bladder carcinoma following low-dose Photofrin injection.. Urology.

[OCR_00843] Campo E., Merino M. J., Liotta L., Neumann R., Stetler-Stevenson W. (1992). Distribution of the 72-kd type IV collagenase in nonneoplastic and neoplastic thyroid tissue.. Hum Pathol.

[OCR_00847] Cortese D. A., Kinsey J. H., Woolner L. B., Payne W. S., Sanderson D. R., Fontana R. S. (1979). Clinical application of a new endoscopic technique for detection of in situ bronchial carcinoma.. Mayo Clin Proc.

[OCR_00857] Cothren R. M., Richards-Kortum R., Sivak M. V., Fitzmaurice M., Rava R. P., Boyce G. A., Doxtader M., Blackman R., Ivanc T. B., Hayes G. B. (1990). Gastrointestinal tissue diagnosis by laser-induced fluorescence spectroscopy at endoscopy.. Gastrointest Endosc.

[OCR_00861] Doiron D. R., Profio E., Vincent R. G., Dougherty T. J. (1979). Fluorescence bronchoscopy for detection of lung cancer.. Chest.

[OCR_00866] Dougherty T. J. (1987). Studies on the structure of porphyrins contained in Photofrin II.. Photochem Photobiol.

[OCR_00873] Grant W. E., Hopper C., MacRobert A. J., Speight P. M., Bown S. G. (1993). Photodynamic therapy of oral cancer: photosensitisation with systemic aminolaevulinic acid.. Lancet.

[OCR_00890] Harris D. M., Werkhaven J. (1987). Endogenous porphyrin fluorescence in tumors.. Lasers Surg Med.

[OCR_00894] Hung J., Lam S., LeRiche J. C., Palcic B. (1991). Autofluorescence of normal and malignant bronchial tissue.. Lasers Surg Med.

[OCR_00899] Kennedy J. C., Pottier R. H. (1992). Endogenous protoporphyrin IX, a clinically useful photosensitizer for photodynamic therapy.. J Photochem Photobiol B.

[OCR_00904] Kennedy J. C., Pottier R. H., Pross D. C. (1990). Photodynamic therapy with endogenous protoporphyrin IX: basic principles and present clinical experience.. J Photochem Photobiol B.

[OCR_00910] Kessel D. (1989). Determinants of photosensitization by purpurins.. Photochem Photobiol.

[OCR_00916] Lam S., Palcic B., McLean D., Hung J., Korbelik M., Profio A. E. (1990). Detection of early lung cancer using low dose Photofrin II.. Chest.

[OCR_00919] Lohmann W., Hugo F. (1989). The effect of NADH on different human and mouse cell lines.. Naturwissenschaften.

[OCR_00923] Lohmann W., Mussmann J., Lohmann C., Künzel W. (1989). Native fluorescence of unstained cryo-sections of the cervix uteri compared with histological observations.. Naturwissenschaften.

[OCR_00929] Moan J. (1986). Porphyrin photosensitization and phototherapy.. Photochem Photobiol.

[OCR_00933] Nelson J. S., Roberts W. G., Berns M. W. (1987). In vivo studies on the utilization of mono-L-aspartyl chlorin (NPe6) for photodynamic therapy.. Cancer Res.

[OCR_00938] Plus R. (1992). A review of in vivo studies of porphyrins and unexpected fluorescences. An interpretation of the results.. Med Hypotheses.

[OCR_00945] Poulsom R., Pignatelli M., Stetler-Stevenson W. G., Liotta L. A., Wright P. A., Jeffery R. E., Longcroft J. M., Rogers L., Stamp G. W. (1992). Stromal expression of 72 kda type IV collagenase (MMP-2) and TIMP-2 mRNAs in colorectal neoplasia.. Am J Pathol.

[OCR_00950] Profio A. E., Sarnaik J. (1984). Fluorescence of HpD for tumor detection and dosimetry in photoradiation therapy.. Prog Clin Biol Res.

[OCR_00956] Razum N., Balchum O. J., Profio A. E., Carstens F. (1987). Skin photosensitivity: duration and intensity following intravenous hematoporphyrin derivates, HpD and DHE.. Photochem Photobiol.

[OCR_00963] Reddi E., Segalla A., Jori G., Kerrigan P. K., Liddell P. A., Moore A. L., Moore T. A., Gust D. (1994). Carotenoporphyrins as selective photodiagnostic agents for tumours.. Br J Cancer.

[OCR_00976] Richter A. M., Cerruti-Sola S., Sternberg E. D., Dolphin D., Levy J. G. (1990). Biodistribution of tritiated benzoporphyrin derivative (3H-BPD-MA), a new potent photosensitizer, in normal and tumor-bearing mice.. J Photochem Photobiol B.

[OCR_00969] Richter A. M., Kelly B., Chow J., Liu D. J., Towers G. H., Dolphin D., Levy J. G. (1987). Preliminary studies on a more effective phototoxic agent than hematoporphyrin.. J Natl Cancer Inst.

[OCR_00990] Spikes J. D. (1986). Phthalocyanines as photosensitizers in biological systems and for the photodynamic therapy of tumors.. Photochem Photobiol.

[OCR_01005] Svanberg K., Andersson T., Killander D., Wang I., Stenram U., Andersson-Engels S., Berg R., Johansson J., Svanberg S. (1994). Photodynamic therapy of non-melanoma malignant tumours of the skin using topical delta-amino levulinic acid sensitization and laser irradiation.. Br J Dermatol.

[OCR_00996] Svanberg K., Kjellén E., Ankerst J., Montán S., Sjöholm E., Svanberg S. (1986). Fluorescence studies of hematoporphyrin derivative in normal and malignant rat tissue.. Cancer Res.

